# Knowledge and Attitude on Insulin Self-Administration among Type 1 Diabetic Patients at Metu Karl Referral Hospital, Ethiopia

**DOI:** 10.1155/2019/7801367

**Published:** 2019-12-14

**Authors:** Tewodros Yosef

**Affiliations:** Department of Public Health, College of Medicine and Health Sciences, Mizan-Tepi University, Mizan Teferi, Ethiopia

## Abstract

**Background:**

Diabetes mellitus (DM) is a group of metabolic diseases characterized by hyperglycemia resulting from defects in insulin secretion, insulin action, or both. It is a public health problem as the disease is epidemic in both developed and developing counties. Knowledge and attitude of patients regarding insulin self-administration could lead to better management of diabetes and eventually a good quality of life. Despite this, the evidence that showed the knowledge and attitude on insulin self-administration is a substantial deficiency in Ethiopia.

**Objective:**

To assess the level of knowledge, attitude, and associated factors on insulin self-administration among type 1 diabetic patients at Metu Karl Referral Hospital, Ethiopia, in 2019.

**Methods:**

An institutional-based cross-sectional study was conducted among systematically selected 245 type 1 diabetic patients at Metu Karl Referral Hospital, Ethiopia, in January 2019. The data were collected through a face-to-face interview. The collected data were entered using EpiData version 4.2.0.0, cleaned, and analyzed using SPSS version 20. A binary logistic regression model was used. Independent variables with a *P* value of less than 0.05 in the multivariable logistic regression model were considered significant.

**Results:**

Out of 242 type 1 diabetic patients interviewed, 93 (38.4%, 95% CI (32.3%-44.5%)) had good knowledge and 50 (20.7%, 95% CI (15.6%-25.8%)) had favorable attitude on insulin self-administration. The study also found that being unmarried (AOR = 3.59, 95% CI (1.15-11.3), *P* = 0.028), increased educational level (AOR = 3.02, 95% CI (1.36-6.74), *P* = 0.007), and more years of treatment (AOR = 3.70, 95% CI (1.16-11.8), *P* = 0.027) were factors associated with good knowledge on insulin self-administration, whereas being a member of DM association (AOR = 3.57, 95% CI (1.66-7.69), *P* = 0.001) was the only factor associated with favorable attitude on insulin self-administration.

**Conclusion:**

The knowledge and attitude on insulin self-administration among type 1 diabetic patients were substantially low. Diabetes and insulin self-administration education should be imparted by health professionals at each follow-up visit. Besides, strengthening of information, education, and communication (IEC) on the issue of diabetes and insulin self-administration using mass media (television/radio) plays paramount importance.

## 1. Introduction

Diabetes mellitus (DM) is a group of metabolic diseases characterized by hyperglycemia resulting from defects in insulin secretion, insulin action, or both [1]. It is a public health problem as the disease is epidemic in both developed and developing counties. DM is recognized as one of the leading causes of premature illness, death, and disability globally [2]. Its prevalence for all age groups worldwide was estimated to be 2.8% in 2000 and 4.4% in 2030 and projected to rise from 171 million in 2000 to 366 million in 2030 [3]. An estimated 14.2 (9.5-29.4) million people aged 20-79 have diabetes in the sub-Saharan Africa (SSA) region, representing a regional prevalence of 2.1-6.7% [4].

Insulin is one of the oldest valuable antidiabetic medications available and also the most effective agent in dropping hyperglycemia when used in appropriate doses [2, 5]. Type 1 DM (T1DM) patients are treated by multiple-dose insulin injection or continuous subcutaneous insulin infusion. To control the burden, patients need to use insulin therapy as ordered by the health care providers [6, 7]. The insulin injection technique is one of the most common areas with the likelihood of errors [5]. It requires sound knowledge and attitude on self-insulin administration by patients so that they can contribute meaningfully to the management of their lives [2].

Better insulin self-administration is associated with good knowledge and a favorable attitude of a patient on insulin self-administration. Different studies conducted worldwide reported that 52.5% in India [1], 50.3% in Turkey [8], 46% in Nepal [9], 98.7% in Ethiopia [10], 55.3% in Ethiopia [2], and 33.3% in Egypt [11] had good knowledge on insulin self-administration. Regarding attitude, 68.0% in Ethiopia [2], 50.3% in Turkey [8], 98% in Ethiopia [10], and 60.1% in Egypt [11] were found to have favorable attitudes on insulin self-administration. Diabetes knowledge was a significant predictor for attitudes of self-management [12]. The factors that influence knowledge and attitude on insulin self-administration are varied and might include age, sex, marital status, educational status, occupation, urban residence, disease duration, duration of insulin use, and family history of DM [1, 8, 9, 11, 13–15].

Knowledge and attitude of patients regarding insulin self-administration could lead to better management of diabetes and eventually a good quality of life. However, the knowledge and attitude gap exists in type 1 diabetes mellitus management that does not allow patients to independently take their medication to reduce the morbidity and mortality associated with diabetes [2]. Even though these patients in Ethiopia face the high risk of treatment complications [10] like patients elsewhere, the evidence that showed the knowledge and attitude on insulin self-administration is a substantial deficiency. The significance of this study was assessing the level of knowledge, attitude, and associated factors on insulin self-administration among type 1 diabetic patients to address the gap and also to provide opportunities for future studies to fill in the gaps that this study could not address.

## 2. Methods

### 2.1. Study Design, Setting, and Period

An institutional-based cross-sectional study was conducted at Metu Karl Heinz Referral Hospital (MKRH) from January 01 to 30, 2019, which is located in the Oromia Region, Ilu Abbabor Zone, Metu Town, 600 km southwest of Addis Ababa, the capital city of Ethiopia. It is a referral hospital for the region, including Gambella. It is government-run but built by the German NGO “Menschen fuer Menschen.” It is named after the founder of the NGO, Karl Heinz Bohm. Currently, it serves as a teaching and health care providing center for the region. A shortage of internists (internal medicine specialists) compelled a general practitioner to provide the treatment and health counseling of diabetes in MKRH. Since diabetic patients were appointed monthly, health and treatment counseling is given at that time during their visit for a checkup and collecting medication. The health and treatment counseling includes the importance of drug adherence, lifestyle modification (diet selection and having regular physical exercise), and avoiding any injury or trauma in order to prevent gangrene.

### 2.2. Populations

The source population was all insulin self-injecting type 1 diabetic patients who had chronic follow-up visit in Metu Karl Referral Hospital during the study period. While the study population was randomly selected from self-injecting type 1 diabetic patients who fulfill inclusion criteria during the study period. All type 1 diabetic patients aged greater than 18, who had follow-up visits during the study period, were included. Diabetes patients who were severely ill and come with diabetes complication were excluded.

### 2.3. Sample Size Determination

The sample size was determined using a single population proportion formula, with the input of *p* which is the expected proportion of good knowledge (55.3%) and favorable attitude (68%) on insulin self-administration in Mekele, Ethiopia [2], precision level (5%), and 95% confidence interval. The sample sizes computed were 380 and 335 for knowledge and attitude, respectively.

(1) $$\begin{array}{c} n=\frac{{\left(\text{Z }\alpha /2\right)}^{2}p\left(1-p\right)}{d^{2}}, \end{array}$$

where *n* is the sample size, *p* is the expected proportion of knowledge and attitude towards self-administered insulin, *d* is the margin of error (precision level), and *Z* *α*/2 is the reliability coefficient (confidence coefficient).

To maximize the accuracy of the data, the highest value (380) with an expected proportion of good knowledge (55.3%) was used. However, the source population (*N* = 535 patients were taking insulin therapy at the time of data collection) was less than 10,000, then reduction formula was used resulted in 222 smaple size and by adding 10% for non response compensation. The final sample size was 245.

### 2.4. Sampling Technique

Unless there is any disease emergency (complication), every diabetic patient is appointed monthly to have a checkup and collect their monthly medication. Based on the data obtained from chronic follow-up registration books, an average of 18 diabetic patients were seen daily and used as a sampling frame. With this consideration to give each diabetic patient an equal chance of inclusion, the total sample size was divided by thirteen days and resulted in 8 diabetic patients to be studied every day. To identify the potential study participants using a systematic random sampling technique, 18 was divided by 8 to obtain the constant for the sampling interval, which was 2. A random number from one and two was chosen as a random start then it was 1. Hence, every two diabetic patient was studied until the total sample size was obtained.

### 2.5. Data Collection Instrument and Procedures

The attitude and knowledge section of the questionnaire was tested for reliability and validity and was also used in Ethiopia in a similar study [2]. The reliability of the analysis of the collected data was determined using Cronbach's alpha test where the reliability coefficient was found to be significant (Cronbach's alpha: 0.78). The questionnaire was composed of four sections: sociodemographic factors, heath profile, knowledge questions about insulin self-administration, and attitude questions about insulin self-administration. The questionnaire was initially prepared in English and then was translated into the local language by Afaan Oromo and English language experts. To ensure consistency, the Afaan Oromo questionnaire was again translated back to English by a different language expert. The data were collected through a face-to-face interview. To assess the quality (validity and reliability), the questionnaire had been pretested in similar setups before the actual data collection was commenced. Training was given for data collectors and supervisors concerning the objective and process of data collection and discusses the presence of an ambiguous question in the questionnaire.

### 2.6. Study Variables

Dependent variables were knowledge and attitude on insulin self-administration.

Independent variables were sociodemographic factors (age, sex, marital status, educational status, and religion) and health profiles (family history of DM, membership of DM association, and duration of insulin use).

### 2.7. Operational Definitions

*Good knowledge* refers to a person who scores greater than the mean value (≥5 or ≥62.5%) of knowledge-based questions. *Poor knowledge* refers to a person who scores less than the mean value (≤4 or <62.5%) of knowledge-based questions. *Favorable attitude* refers to a person who scores more than the mean value (≥70%) of attitude questions. *Unfavorable attitude* refers to a person who scores less than the mean value (<70%) of attitude questions.

### 2.8. Data Processing and Analysis

The data collected were entered into EpiData version 4.2.0.0, cleaned, and analyzed using SPSS version 20. Binary logistic regression analysis was used to look for an association between outcome and independent variables and dependent variables. Independent variables with a *P* value of less than 0.25 in bivariate logistic regression were included in multivariable logistic regression. Multivariable logistic regression analysis was done to control for potential confounding factors and identify the most important determinate variables. Finally, variables in multivariable logistic regression with a *P* value < 0.05 were considered significantly associated with the outcome variable. Multicollinearity between independent variables in each model was checked, and the variance inflation factor (VIF) was found to be acceptable (less than 2). The Hosmer-Lemeshow goodness of fit test indicated that for knowledge and attitude on insulin self-administration (*P* = 0.785 and *P* = 0.587, respectively), the multivariable logistic regression models were good enough to fit the data well.

## 3. Results

### 3.1. Sociodemographic Characteristics of the Respondents

Out of 245, 242 participated in the study yielding a response rate of 98.7%. The mean age of the respondents was 33.7 (±12.6 SD) years with a range of 19 to 70 years. The majority of 150 (62%) of the respondents were below the mean age (33.7 years). More than half (144 (59.5%)) were females. One hundred eight (44.6%) of the participants were protestant followers. One hundred thirty (53.7%) and 99 (40.9%) of respondents were married and attended secondary and above school, respectively (Table 1).

### 3.2. Health-Related Profile of the Respondents

More than half (59.1%) of the study participants had no family history of diabetes. One hundred forty-one (58.3%) participants were members of the diabetic association. Regarding the duration treatment use, 213 (88.0%) of study participants were taking insulin treatment for more than 5 years. More than three-fourths (80.6%) of the respondents define diabetes correctly as “the presence of high blood sugar.” One hundred fourteen (47.1%) experienced hypoglycemia after injecting insulin. Of those who develop hypoglycemia, 86 (75.4%), 20 (17.6%), and 8 (7.0%) were managed by home treatment using sugar, candy, and honey, respectively.

### 3.3. Knowledge regarding Insulin Self-Administration (ISA)

The mean knowledge score was 4.97 (±1.16 SD) with a range from 2 to 8. Nighty-three (38.4%) respondents had good knowledge on insulin self-administration. One hundred thirty-two (54.5%) correctly answered that insulin is used to lower blood glucose. More than three-fourths (78.9%) answered that insulin injection should be done before taking a meal or just soon after a meal. One hundred sixty-seven (69.0%) correctly answered that sites for insulin injection are the abdomen, thigh, glutei, and deltoid. More than half (56.2%) answered that an insulin vial is stored in the refrigerator or cold place or sand soaked with water. One hundred eighty-five (76.4%) correctly answered that rotation of the injection site is used to reduce pain and prevent wasting of subcutaneous tissues. Two hundred thirty (95.0%) answered that the complications of insulin therapy are low blood sugar, insulin allergy, insulin resistance, and wasting of subcutaneous tissue (Table 2).

### 3.4. Attitude regarding Insulin Self-Administration

The mean attitude score of respondents was 17.7 (±2.93 SD) with a range of 7 to 25. Fifty (20.7%) respondents had a favorable attitude on insulin self-administration. Seventy-four (30.6%) participants agreed that insulin causes other health problems. More than half (62.0%) of the study subjects agreed that insulin self-administration correctly decreases glucose in the blood. One hundred seventy (48.3%) of study subjects agreed that insulin self-administration is tiresome, and ninety-three (38.4%) of respondents disagreed that insulin self-administration does not bring stigma. Seventy-seven (31.8%) participants agreed that insulin self-administration is beneficial regarding cost and time (Table 3).

### 3.5. Bivariate and Multivariable Analyses

The association of each independent variable with knowledge and attitude on insulin self-administration was tested using binary logistic regression analysis. Independent variables found to be statistically significant at *P* < 0.25 in the bivariate analysis were included in the multivariable binary logistic regression model.

In bivariate analysis, age group, marital status, educational status, diabetes association membership, and duration of insulin use were statistically associated with good knowledge on ISA at *P* value < 0.25 and finally marital status, educational status, and duration of insulin use were found to be significantly associated with good knowledge on ISA at *P* value < 0.05 in the multivariable logistic regression model (Table 4).

In bivariate analysis, age group, marital status, educational status, and membership of diabetes association were statistically associated with a favorable attitude on ISA at *P* value < 0.25 and finally membership of diabetes association was found to be significantly associated with a favorable attitude on ISA at *P* value < 0.05 in the multivariable logistic regression model (Table 5).

## 4. Discussion

This study was aimed at assessing knowledge and attitude on insulin self-administration and factors associated with type 1 diabetic patients. As a result, the proportion of good knowledge on insulin self-administration was found to be 38.4% (32.3%-44.5%). This study was in line with 33.3% of the patients in a study in Egypt [11]. It was lower than 52.5% in India [1], 46% in Nepal [9], 50.3% in Turkey [8], and 70.4% in Tigray, Ethiopia [16]. And the proportion of favorable attitude on insulin self-administration was found to be 20.7% (15.6%-25.8%). It was lower than 68.0% in Mekele, Ethiopia [1], 60.1% in Egypt [11], and 70.4% in Tigray, Ethiopia [16]. The variation observed compared to other studies could be due to the differences in sample size, the operational definition used, and the methodology in general. Besides, the socioeconomic, cultural, and educational profile of the study population may create a significant variation between studies.

In this study, 33.5% (81) of the respondents were single. Marital status was found to be associated with knowledge on insulin self-administration. Being single was strongly associated with the level of knowledge. This means that those who are single had 3.6 times increased odds of having good knowledge on insulin self-administration than those who are married and divorced/widowed. The finding was consistent with a study conducted in Gondar, Ethiopia [15].

Ninety-nine (40.9%) of the respondents achieved secondary and above educational level. As a result, respondents who achieved secondary school and above had 3 times increased odds of having good knowledge on insulin self-administration than primary school achiever and below. An increased educational level was strongly associated with the level of knowledge. The finding was consistent with a study conducted in different countries [1, 5, 13, 17]. It may be due to having good educational status correlated with having good knowledge with diseases, disease treatment, importance, practice, and adherence to treatments.

Out of 242, 88% (213) of the respondents used insulin for five or more years. Those who use insulin for five or more years had 3.7 times increased odds of having good knowledge on insulin self-administration than those who use insulin for less than five years. More years of taking insulin were strongly associated with the level of knowledge. The finding was consistent with a study conducted in India and Ethiopia [5, 18]. This might be due to the idea that the more they use, the better they know it.

More than half (58.3%) of the respondents were members of the Ethiopian diabetes association. Those who are members of diabetes association had 3 times increased odds of having a favorable attitude on insulin self-administration than those who are not. Being a member of the diabetes association was strongly associated with a favorable attitude and adherence to insulin use [6]. It could be because those who are members of the diabetes association might have the chance to get awareness creation services organized by the association; this may change their level of attitude as a result of their good knowledge.

### 4.1. Limitation of the Study

This study assessed only knowledge and attitude on insulin self-administration. But the actual practice of insulin self-administration, whether it is good or poor practice, was not assessed. The possibility of social desirability bias may be considered a limitation to this study for participants who were interviewed upon their arrival to the chronic follow-up clinic of the hospital. This may shadow their responses to interview questions.

## 5. Conclusion

The knowledge and attitude on insulin self-administration among type 1 diabetic patients were substantially low. Diabetes and insulin self-administration education must be imparted by health professionals at each follow-up visit. Besides, strengthening of information, education, and communication (IEC) on the issue of diabetes and insulin self-administration using mass media (television/radio) plays paramount importance.

## Figures and Tables

**Table 1:** Sociodemographic characteristics of type one diabetic patients at MKRH, 2019.

Variables		Frequency	Percent
Age group	<33.7 years	150	62.0
	≥33.7 years	92	38.0

Sex	Male	98	40.5
	Female	144	59.5

Religion	Protestant	108	44.6
	Orthodox	75	31.0
	Muslim	59	24.4

Marital status	Single	81	33.5
	Married	130	53.7
	Divorced/widowed	31	12.8

Educational status	Unable to read and write	58	24.0
	Able to read and write to G-8	85	35.1
	Secondary and above	99	40.9

**Table 2:** Knowledge regarding ISA among type 1 diabetic patients at MKRH, 2019.

Items	Correct answer	Wrong answer
	Frequency (%)	Frequency (%)
Insulin is used to lower blood glucose	132 (54.5%)	112 (45.5%)
Insulin injection is taken before or just soon after a meal	191 (78.9%)	51 (21.1%)
The sites for insulin injection are the abdomen, thigh, glutei, and deltoid	167 (69.0%)	77 (31.0%)
An insulin vial is stored in the refrigerator or cold place or sand soaked with water	136 (56.2%)	108 (43.8%)
The use of the rotation of the injection site is to reduce pain and prevent wasting of subcutaneous tissues	185 (76.4%)	59 (23.6%)
Massage after injection is used to reduce the rapid absorption of insulin	17 (6.2%)	227 (93.8%)
The complications of insulin therapy are low blood sugar, insulin allergy, insulin resistance, and wasting of subcutaneous tissue	230 (95.0%)	12 (5.0%)
The benefits of insulin self-administration are that it is time-saving, cheap, and easily portable	190 (78.5%)	54 (21.5%)

**Table 3:** Attitude regarding ISA among type 1 diabetic patients at MKRH, 2019.

Questions	Strongly disagree	Disagree	Not sure	Agree	Strongly agree
Insulin causes other health problems	24 (9.9%)	75 (31.0%)	35 (14.5%)	74 (30.6%)	34 (14%)
ISA correctly decreases glucose in the blood	2 (0.8%)	19 (7.9%)	23 (9.5%)	150 (62%)	48 (19.8%)
ISA is tiresome	4 (1.7%)	22 (9.1%)	23 (9.5%)	76 (31.4%)	117 (48.3%)
ISA brings stigma	30 (12.4%)	93 (38.4%)	21 (8.7%)	77 (31.8%)	21 (8.7%)
ISA is beneficial regarding cost and time	45 (18.6%)	51 (21.1%)	5 (2.1)	77 (31.8%)	64 (26.4%)

**Table 4:** Bivariate and multivariable logistic regression analyses of factors associated with good knowledge on ISA among type 1 diabetic patients at MKRH, 2019.

Variables	Categories	Knowledge on ISA		COR (95% CI)	AOR (95% CI)	*P* value
		Poor	Good			
Age group	<33.7 years	87	63	1	1	
	≥33.7 years	62	30	0.67 (0.39-1.15)	0.91 (0.49-1.68)	0.751

Marital status	Single	45	36	4.65 (1.54-14.1)	3.59 (1.15-11.3)	0.028^∗^
	Married	69	53	5.40 (1.73-16.8)	2.96 (0.86-10.1)	0.084
	Divorced/widowed	27	8	1	1	

Educational status	Unable to read and write	43	15	1	1	
	Able to read and write to G-8	61	24	1.13 (0.53-2.40)	1.20 (0.53-2.70)	0.667
	Secondary and above	45	54	3.44 (0.78-3.17)	3.02 (1.36-6.74)	0.007^∗∗^

Diabetes association membership	Yes	78	63	1.91 (1.11-3.28)	1.44 (0.78-2.66)	0.243
	No	71	30	1	1	

Duration of insulin use	<5 years	25	10	1	1	
	≥5 years	124	83	4.49 (1.51-13.3)	3.70 (1.16-11.8)	0.027^∗^

CI = confidence interval; COR = crude odds ratio; AOR = adjusted odds ratio; ∗ = significant at *P* value < 0.05; ∗∗ = significant at *P* value < 0.01; ISA = insulin self-administration.

**Table 5:** Bivariate and multivariable logistic regression analyses of factors associated with a favorable attitude on ISA among type 1 diabetic patients at MKRH, 2019.

Variables	Categories	Attitude favorability of ISA		COR (95% CI)	AOR (95% CI)	*P* value
		No	Yes			
Age group	<33.7 years	126	24	1	1	
	≥33.7 years	66	26	2.07 (1.10-3.88)	1.88 (0.95-3.75)	0.072

Marital status	Single	70	11	0.73 (0.31-1.76)	0.71 (0.28-1.83)	0.481
	Married	100	30	0.38 (0.14-1.05)	0.66 (0.22-2.02)	0.466
	Divorced/widowed	22	9	1	1	

Educational status	Unable to read and write	41	17	1	1	
	Able to read and write to G-8	66	19	0.69 (0.32-1.49)	0.89 (0.39-2.03)	0.783
	Secondary and above	85	14	0.40 (0.18-0.88)	0.48 (0.19-1.18)	0.108

Diabetes association membership	Yes	102	39	3.13 (1.51-6.74)	3.57 (1.66-7.69)	0.001^∗∗^
	No	90	11	1	1	

CI = confidence interval; COR = crude odds ratio; AOR = adjusted odds ratio; ∗∗ = significant at *P* value < 0.01.

## Data Availability

The data set is handled by the corresponding author and can be provided upon request.

## Abbreviations

AOR: Adjusted odds ratio
CI: Confidence interval
COR: Crude odds ratio
DM: Diabetes mellitus
G-8: Grade 8
ISA: Insulin self-administration
MKRH: Metu Karl Referral Hospital
SPSS: Statistical Package for the Social Sciences
SD: Standard deviation.
